# Hormone Replacement Therapy Is Associated with Disease Activity Improvement among Post-Menopausal Women with Inflammatory Bowel Disease

**DOI:** 10.3390/jcm13010088

**Published:** 2023-12-23

**Authors:** Morgan Freeman, Lauren Lally, Levi Teigen, Elliot Graziano, Raina Shivashankar, Eugenia Shmidt

**Affiliations:** 1 Division of Gastroenterology, Hepatology, and Nutrition, University of Minnesota, Minneapolis, MN 55455, USA; mensi026@umn.edu (M.F.); teige027@umn.edu (L.T.); elliot.graziano@parknicollet.com (E.G.); 2 Division of Gastroenterology and Hepatology, Thomas Jefferson University, Philadelphia, PA 19107, USAraina.shivashankar@jefferson.edu (R.S.)

**Keywords:** inflammatory bowel disease, hormone replacement therapy, menopause

## Abstract

(1) Background: There are limited data available to guide clinical decision-making regarding the effects of hormone replacement therapy (HRT) in post-menopausal women with inflammatory bowel disease (IBD). In this study, we sought to characterize a population of post-menopausal women with IBD and to determine the effects of HRT on their disease activity. (2) Methods: A multicenter, retrospective, case–control cohort study of post-menopausal women with IBD was conducted. The physician global assessment (PGA) score was used to quantify disease activity. To control for the effects of menopause, IBD patients who had not undergone HRT were used as controls. (3) Results: There was a significant reduction in the frequency of PGA scores ≥2 post HRT treatment (*p* < 0.01). HRT treatment was associated with a 5.6× increase in the odds of post-HRT PGA score improvement compared to controls (OR 5.6; 95% CL 1.6, 19.7) in our univariate logistic regression analysis. (4) Conclusion: Post-menopausal IBD women who underwent HRT therapy showed an improvement in their disease symptoms following HRT compared to post-menopausal women without HRT therapy, who showed no change.

## 1. Introduction

Inflammatory bowel disease (IBD) is a chronic condition caused by an inappropriate immune response in the gastrointestinal tract. Its cause, while not well understood, is likely multifactorial, with possible contributions including genetic factors, fecal microbiota properties, gut epithelial characteristics, and immune properties [1]. Previous studies have observed changes in IBD symptoms during times of hormone fluctuation, such as menstrual periods and pregnancy [2,3]. These same studies have not found a significant change in symptoms during menopause. However, there are conflicting data on the use of hormone replacement therapy in menopause and its effect on disease activity.

Sex hormones such as estrogen are thought to play a role in modulating inflammation in the gastrointestinal tract [4]. Regarding the use and effects of exogenous hormones on inflammatory bowel disease, the current literature is most robust in the area of oral contraceptive pill (OCP) use in premenopausal IBD patients. Oral contraception has not been associated with an increased risk of IBD activity. [5,6] The implications of reproductive health and the use of oral contraceptive pills (OCPs) have been widely studied. However, there has been little evidence to characterize the impact of hormone replacement therapy (HRT) in post-menopausal women.

At this time, there are limited data to guide clinical decision-making regarding the safety and efficacy of HRT use in this population. This gap in research and knowledge may make it difficult for clinicians to counsel patients on the possible effects of hormone replacement therapy for menopause symptoms. Many females will experience vasomotor activity and other symptoms related to menopause. With sparse data available to make informed decisions, patients may forego treatment of their menopause symptoms to avoid the unknown risks concerning how it may affect their IBD activity. We sought to build from existing research and add to this important area of care for females with inflammatory bowel disease.

We hypothesized that post-menopausal women initiated on hormone replacement therapy would experience an improvement in the disease activity of their inflammatory bowel disease. We aimed to conduct a multicenter retrospective cohort study to describe post-menopausal women with IBD and to determine the effects that HRT may have on disease activity.

## 2. Materials and Methods

### 2.1. Patients

We performed a retrospective case–control cohort study of post-menopausal women with IBD seen at the University of Minnesota and Thomas Jefferson University Hospital from 1 January 2000 to 1 January 2020. Electronic health records were manually reviewed to confirm IBD diagnosis and subtype (Crohn’s disease (CD) or ulcerative colitis (UC)). Menopause was defined by either natural menopause (>/=12 months of amenorrhea) or surgical menopause (hysterectomy and bilateral oophorectomy).

### 2.2. Selection of Controls

To control for the effects of menopause on disease activity, post-menopausal women with IBD who had not undergone HRT were identified via chart review and used as controls. Patients were matched by age at menopause onset ±5 years and subtype of IBD. Disease activity of patients before and after menopause at any time was collected in the control group.

### 2.3. Protocol

Data were obtained from a manual review of medical records. IBD activity was quantified using the physician global assessment (PGA) score per clinical documentation closest to the time point of interest (i.e., onset of HRT therapy for the HRT group and menopause for the control group). The PGA score was determined via chart review with one individual at each participating institution assessing patient charts. After reviewing their clinical documentation, along with recent laboratory and endoscopic results, each patient’s disease activity was rated as in remission (PGA score = 0), mild (PGA score = 1), moderate (PGA score = 2), or severe (PGA score = 3). Based on the clinician’s chart review of clinical notes and objective data, remission was defined as a patient with no symptoms attributed to active IBD. Mild disease activity was defined as a patient with active IBD symptoms that did not require a change of medication or dose escalation. Moderate disease activity was defined as a patient with symptoms requiring escalation of IBD therapy of either dose, class of medication, or oral steroids. Severe disease activity was defined as a patient with symptoms requiring hospitalization, surgery, or intravenous steroids.

Data collected for patients who received hormone replacement therapy included patient demographics, age at menopause onset, type of menopause, IBD history, duration of disease, and IBD-related hospitalizations and surgeries. Information regarding IBD therapy at the time of HRT initiation, as well as IBD therapy escalation and IBD flares post HRT, was collected. The control patients’ charts were reviewed for disease activity pre and post-menopause using the PGA score and IBD-related surgery before and after menopause; for patients who received HRT, the type of HRT (estrogen monotherapy or estrogen plus progesterone) was determined.

### 2.4. Statistical Analysis

All statistical analyses were performed using SAS version 9.4 (SAS Institute, Cary, NC, USA). *p* values < 0.05 were considered significant. Missing values were excluded from the analyses. Descriptive statistics (mean and standard deviation for continuous variables or frequency for categorical variables) were used to assess the characteristics of patients initiated on HRT and control patients. T-tests were used to compare continuous variables between patients started on HRT and the controls, and Pearson’s chi-squared test (for values > 5) or Fisher’s exact test (for values < 5) were used to compare categorical variables.

McNemar’s test was used to compare outcomes pre and post HRT, given the paired nature of these data. This analysis only included patients initiated on HRT, which allowed for an increased cohort size to 37 (vs. 31 in the matched analysis) because it was not limited to those with a matched control.

## 3. Results

We identified 37 patients with IBD who were post-menopausal and initiated on HRT. The mean age at the onset of menopause in the patients initiated on HRT was 46 years (range: 25–66); 59% had Crohn’s disease (CD), and 41% had ulcerative colitis (UC) (Table 1). The average duration of IBD before menopause was 12.7 years (range: 0.5–32 years). The type of IBD treatment at the time of HRT initiation and the type of HRT are listed in Table 1. The majority (70%) of patients initiated on HRT were on estrogen therapy alone, and 30% were on a combined estrogen and progesterone HRT regimen. 

### 3.1. Pre- and Post-HRT Outcomes

Overall, 9 of the 37 (24%) patients required an escalation in their therapy at any time in the one-year period after HRT initiation, with an average time until escalation of 10.5 months. One patient required steroids for an IBD flare, three had their current therapy dose or frequency increased, and five patients required a change to their IBD therapy. A significant reduction in frequency of PGA scores ≥ 2 (moderate to severe disease activity) was observed when comparing pre- and post-HRT treatment scores (*p* < 0.01; Table 2).

### 3.2. Analysis with Matched Controls

Controls (*n* = 31) were successfully matched to the HRT patients according to age and IBD type (Table 3). Using the physician global assessment (PGA) to determine disease activity, 71% of the matched HRT cohort (*n* = 31) had active disease in the premenopausal period compared to 39% in the control group. Based on the PGA scores, there was greater disease severity in the HRT cohort pre menopause (*p* = 0.03). There was no significant difference in disease activity between the HRT cohort and the controls post menopause. There was a significant reduction in the frequency of PGA scores ≥ 2 (moderate to severe disease activity) post HRT treatment (*p* < 0.01). HRT treatment was associated with a 5.6× increase in the odds of a post-HRT PGA score improvement compared to the controls (OR 5.6; 95% CL 1.6, 19.7) in our univariate logistic regression analysis.

In the premenopausal period, 14 patients (45%) in the matched HRT cohort underwent IBD-related surgery compared to 10 patients (32%) in the controls. In the post-menopausal period, four patients (13%) in the matched HRT vs. zero patients (0%) in the controls required IBD-related surgery. There was no statistically significant differences in IBD-related surgery between the matched groups in the pre- or post-menopausal period (Table 1).

## 4. Discussion

In this multicenter, retrospective cohort study, we aimed to identify a population of post-menopausal women with IBD and to determine the effect that HRT may have on disease activity. We found our hypothesis to be true that post-menopausal women with IBD who underwent hormone replacement therapy showed an improvement in their disease activity based on physician global assessment scores following HRT compared to post-menopausal women with IBD who were not on HRT. Pre-menopausal disease activity seemed to be higher in patients who underwent HRT compared to the controls. We found that women with a more active disease were more likely to undergo HRT, which may be because women with active IBD have been shown to report more clinically significant symptoms related to menopause [7].

To date, there has been one other study that investigated the effects of HRT on IBD activity in the post-menopausal period using primary medical records [2]. This was a retrospective, single-institution study from over a decade ago that did not have a separate control group [2]. Kane et al. found a protective effect of HRT on disease activity in post-menopausal women with IBD. In this study, post-menopausal women on HRT were 80% less likely to have an IBD flare [8]. More recently, Rolston and colleagues evaluated the influence of hormonal fluctuation on IBD symptoms and noted that of patients undergoing HRT for menopause symptoms, 61% reported no change in their IBD symptoms with HRT [3]. Of note, no post-menopausal patients reported a worsening of their symptoms while using HRT in the study.

There are physiological mechanisms that may explain our results. Endogenous sex hormone fluctuations and their influence on the gastrointestinal tract in patients with and without IBD have been established [2,3,9]. The estrogen surge during the menstrual cycle has been shown to impact gut permeability, transit time, and pain perception [10]. The gastrointestinal tract contains estrogen receptor alpha and estrogen receptor beta, which play different roles in estrogen signaling; estrogen receptor beta is the primary receptor in colon tissue [9]. In mice models, treatment with 17β-estradiol was associated with reduced inflammation in the colon, and mice with knocked-out estrogen receptor beta had increased colon inflammation [11,12]. Exogenous sex hormones, such as those used in hormone replacement therapy, likely exhibit similar anti-inflammatory effects in the gut.

### 4.1. Limitations

There are several limitations to our study. Objective data for the disease activity assessment, such as inflammatory markers or endoscopic findings, were limited in our patient cohort. Using the physician global assessment (PGA) requires the judgment of disease activity by a clinician via chart reviews, which can introduce bias and variability. We attempted to limit this by designating one provider at each participating institution for the chart review. Additionally, the PGA score only allows for a general assessment of overall symptoms, and we were unable to report changes in specific symptoms such as stool frequency, stool consistency, or abdominal pain. Future studies should aim to include more objective data on disease activity, as well as additional details of IBD care such as disease phenotype and indication for surgery. The physician global assessment data were collected from the clinical visit closest to the onset of HRT or menopause. This is another limitation, as we were unable to control for the effect that time may or may not have had on disease activity. Lastly, the sample size is likely underpowered to make our findings generalizable, since we included patients with both ulcerative colitis and Crohn’s disease, as well as on variable therapies. In addition, we were unable to obtain matched controls for the full HRT cohort.

### 4.2. Strengths

Given that there is a paucity of information on this topic, our study adds to previous reports which have shown a protective effect of HRT on IBD activity [8]. We used age-matched controls to adjust for the effects of menopause on IBD activity, making the present study the first to do so for this specific question. Despite the relatively smaller sample size, our analysis provides valuable information to clinicians and patients regarding the effects of HRT medications on inflammatory bowel disease activity in post-menopausal women. Moreover, our study includes patients with both Crohn’s disease and ulcerative colitis from different institutions, which is likely reflective of real-world clinical practices. 

In conclusion, we found that the use of HRT was associated with improvements in inflammatory bowel disease symptoms in post-menopausal women. In an area that is understudied and challenged with mixed data, we hope that our findings help with counseling patients on the anticipated impact of HRT on IBD activity, especially for patients with a more severe disease. Future studies with a prospective design, larger sample size, and objective disease activity measurements are needed to confirm our findings.

## Figures and Tables

**Table 1:** Patient characteristics of patients initiated on HRT.

	Full HRT Cohort (*n* = 37)
Age at menopause onset, years	46.4 ± 9.0
Menopause type:	
Natural	26 (70%)
Surgical	11 (30%)
IBD type:	
Crohn’s	22 (59%)
Ulcerative colitis	15 (41%)
Duration of IBD, years	12.7 ± 10.5
IBD treatment at HRT initiation:	
None	10 (27%)
5-ASA	9 (24%)
Immunomodulator	1 (3%)
Biologic	4 (11%)
Biologic and immunomodulator	13 (35%)
Type of HRT:	
Estrogen alone	26 (70%)
Estrogen + progesterone	11 (30%)

**Table 2:** Outcomes pre and post hormone replacement therapy.

	Pre HRT	Post HRT	*p*-Value
Hospitalized (*n* = 25)	10 (40%)	8 (32%)	0.32
Surgery	17 (46%)	5 (14%)	<0.01
PGA scores:			<0.05
Remission	9 (24%)	17 (46%)	
Mild	16 (43%)	17 (46%)	
Moderate	7 (19%)	1 (3%)	
Severe	5 (14%)	2 (5%)	
Moderate to severe disease	12 (32%)	3 (8%)	<0.01

**Table 3:** Characteristics of patients initiated on HRT and matched controls.

	Matched HRT Cohort (*n* = 31)	Controls (*n* = 31)	*p*-Value (Matched HRT vs. Control)
Age at menopause	49.2 ± 6.5	48.3 ± 5.3	NS
Menopause type:			
Natural	19 (61%)	NA	
Surgical	12 (39%)	NA	
IBD type:			NS
Crohn’s	19 (61%)	19 (61%)	
Ulcerative colitis	12 (39%)	12 (39%)	
Duration of IBD	12.8 ± 10.9	NA	
Type of HRT:			
Estrogen alone	20 (65%)	NA	
Estrogen + progesterone	11 (35%)	NA	
Disease severity pre menopause:			0.03
0	9 (29%)	19 (61%)	
1	13 (42%)	9 (29%)	
2	5 (16%)	3 (10%)	
3	4 (13%)	0 (0%)	
Disease severity post menopause:			NS
0	17 (55%)	21 (68%)	
1	12 (39%)	7 (23%)	
2	1 (3%)	3 (10%)	
3	1 (3%)	0 (0%)	
PGA ≥ 2 pre menopause			NS (0.11)
No	22 (71%)	28 (90%)	
Yes	9 (29%)	3 (10%)	
PGA ≥ 2 post menopause			NS
No	29 (94%)	28 (90%)	
Yes	2 (6%)	3 (10%)	
Surgery pre menopause			NS
No	17 (55%)	21 (68%)	
Yes	14 (45%)	10 (32%	
Surgery post menopause			NS
No	27 (87%)	31 (100%)	
Yes	4 (13%)	0 (0%)	

## Data Availability

Research data available by request.

## Abbreviations

HRT	Hormone replacement therapy
IBD	Inflammatory bowel disease
PGA	Physician global assessment
OD	Odds ratio
CL	Confidence level
OCP	Oral contraceptive pill
CD	Crohn’s disease
UC	Ulcerative colitis
NC	North Carolina
USA	United States of America
